# The stigma of patients with chronic insomnia: a clinical study

**DOI:** 10.1186/s12888-022-04091-y

**Published:** 2022-07-05

**Authors:** Shuo He, Xue-Jia Ke, Yan Wu, Xiao-Yi Kong, Yun Wang, Hui-Qin Sun, Deng-Zhi Xia, Gui-Hai Chen

**Affiliations:** 1 Department of Neurology (Sleep Disorders), The Affiliated Chaohu Hospital of Anhui Medical University, Hefei, 238000 Chaohu China; 2 Department of Geriatrics, Hefei First People’s Hospital, Hefei, 230092 China; 3 Department of Outpatient, The Affiliated Chaohu Hospital of Anhui Medical University, Hefei, 238000 Chaohu China

**Keywords:** Chronic insomnia disorder, Health status, Mental health, Quality of life, Stigma

## Abstract

**Background:**

The objective of this study was to explore the stigma and related influencing factors in individuals with chronic insomnia disorder (CID).

**Methods:**

A total of 70 CID patients and 70 healthy controls (CON) were enrolled in the study. All subjects completed the assessments of sleep, emotion, and cognition. Their stigma and life quality were measured using the Chronic Stigma Scale and the 36-Item Short-Form Health Survey (SF-36).

**Results:**

The ratio of individuals with stigma was significantly different between CID and CON groups (C^2^ = 35.6, *p* < 0.001). Compared with the CON group, the CID group had higher scores for total stigma (U = 662.0, *p* < 0.001), internalized stigma (U = 593.0, *p* < 0.001), enacted stigma (U = 1568.0, *p* < 0.001), PSQI (U = 2485.0, *p* < 0.001) and HAMD-17 (U = 69.5, *p* < 0.001) as well as lower scores for MoCA-C (U = 3997.5, *p* < 0.001) and most items of SF-36. Partial correlation analysis showed that different items of the Chronic Stigma Scale were positively correlated with illness duration, PSQI and HAMD-17 scores, while negatively correlated with one or more items of the SF-36. Multivariate regression analysis showed that illness duration and the Mental Health domain of the SF-36 were independent risk factors for one or more items of stigma in CID patients.

**Conclusion:**

Patients with CID have an increased risk of stigma. Moreover, illness duration and Mental Health may be primary factors related to stigma.

**Supplementary Information:**

The online version contains supplementary material available at 10.1186/s12888-022-04091-y.

## Introduction

In the general population, insomnia is a common health problem, with 19% to 50% of adults having sleep complaints [1]. Roughly half of insomnia patients who fulfill the diagnostic for for insomnia disorder have a chronic course [2–6]. As a common accompanying symptom of insomnia, depression can aggravate the severity of insomnia, highlighting the importance of evaluating depression severity in insomnia patients [7, 8]. Additionally, long-term insomnia could lead to cognitive dysfunction that displays clinically significant impairment of performance [9]. Increased impaired work productivity, reduced quality of life, mental health problems, and socioemotional difficulties are also common in those with insomnia [10–13]. Accordingly, there is an urgent need to explore related influence factors of chronic insomnia disorder (CID), which would inform future attempts to alleviate or treat insomnia symptoms.

Stigma manifests as both prejudice of and rejection from society towards patients who suffer from a specific pathology [14]. A patient’s internalization of such discrimination can have repercussions on their state of mind and quality of life [14]. It is possible to distinguish between “enacted stigma”, which consists of actual discrimination and rejection by society towards the patient, and “internalized stigma”, whereby the patient becomes aware of the discrimination and internalizes the negative impact of their disease [15]. Different degrees of stigma seriously affect physical health conditions, which can subsequently exacerbate mental health conditions and further harm an individual’s health [16]. Additionally, psychiatric disorders have been reported to accompany stigmatization, which results in increased psychological distress and decreased health-related quality of life [17].

Misperceptions of sleep disorders also perpetuate stigma and are labeled by others as antisocial, lazy, or faking [18, 19]. Additionally, people who have poorer global sleep quality and more daytime dysfunction are commonly accompanied with tired facial expressions and decreased perception of performance in the workplace [20, 21]. While such impaired functioning plays an important driver for help-seeking behavior [4], not all individuals seek professional treatment after suffering from insomnia [22]. On the one hand, the participants’ perception of insomnia as benign, trivial, or a problem one should be able to cope with alone [23]. Additionally, some of them are afraid their insomnia will be misunderstood or not taken seriously by friends or family members [23]. In research about understanding determinants of insomnia patients whose help-seeking behavior, stigma is thought to be one of common barriers to treatment seeking [23, 24]. Meanwhile, the most common reasons cited for delay centered around considerable social stigma patients felt about having sleeping problems [24]. Thus, while the exist of stigma influence the treatment of insomnia, there is limited research examining stigma in insomnia. Moreover, it is unclear whether CID has a distinct relationship with stigma.

This study aimed to better understand: 1) the risk of stigma in patients with CID and 2) related influencing factors of stigma in CID patients. Our study may contribute to the knowledge gap regarding chronic insomnia stigma and offer a better understanding of the experiences of individuals with sleep disorders who experience stigma.

## Methods

### Subjects

**Fig. 1:**
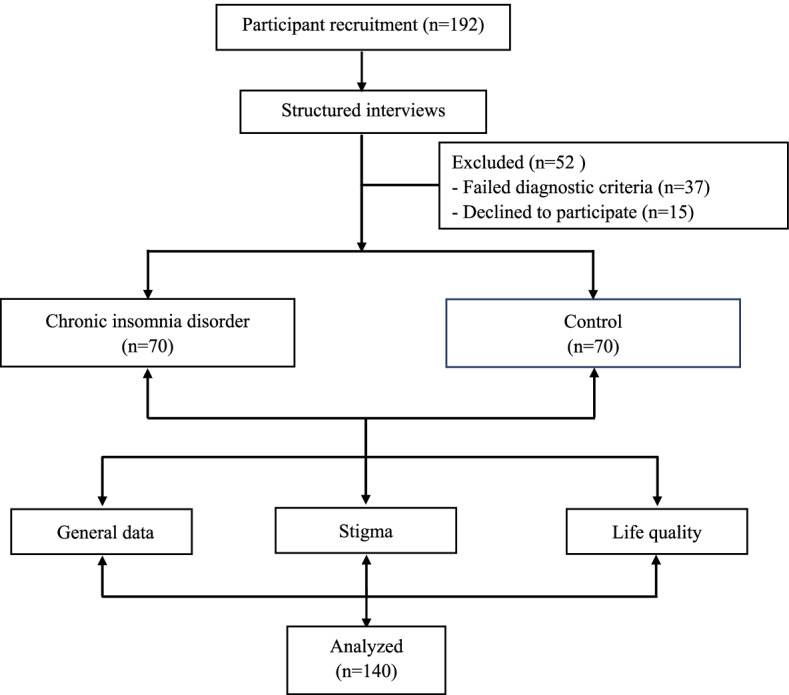
Flowchart of the study participants

A total of 70 CID patients were recruited from the Clinic of Sleep and Memory Disorders in the Affiliated Chaohu Hospital of Anhui Medical University from September 2019 to June 2020. According to preliminary calculation, our sample sizes were accorded with α = 0.05, 1 − β > 0.6, OR = 1.5 by Power software. The flowchart of the study participants is presented in Fig. 1. To ensure the consistency of enrolled participants and whether they met the inclusion criteria, all participants received structured interview according to the Chinese version of Mini International Neuropsychiatry Interview 5.0 (Mini 5.0) by Master’s and doctoral-level evaluators with background and training in psychiatry [25]. In addition to meeting the International Classification of Sleep Disorders, Third Edition (ICSD-3) diagnostic criteria for CID [26], inclusion criteria for patients were as follows: (1) aged between 18 and 75 years; (2) had at least 6 years of education without problems in comprehension; (3) not taking drugs that could potentially interfere with sleep, cognitive function or endocrine function in the 3 months prior to enrolment; and (4) voluntarily participating in the study after providing written informed consent. Exclusion criteria were as follows: (1) somatic comorbidity (including immunologic, endocrine, cardiovascular, neurologic, liver, kidney or organic brain disease); (2) history of substance abuse; (3) recent infection or inflammation (within 2 weeks of the start of the study); (4) taking drugs that could affect sleep, mood, immune function or cognition; and (5) pregnant or lactating women.

At physical examination centers of the same hospital, we also recruited 70 healthy controls (CON) based on similar background information to that of the experimental group (Pittsburgh Sleep Quality Index [PSQI] and 17-Item Hamilton Depression Rating Scale [HAMD-17] scores < 7 [27–30]; a score of ≥ 26 on the Chinese-Beijing Version of Montreal Cognitive Assessment [MoCA-C] [31]; and no insomnia or related medical history during the same period). The study was approved by the Affiliated Chaohu Hospital of Anhui Medical University Ethics Committee (approval no. 201805-kyxm-01).

### General data collection

General information was collected using a questionnaire, which included sex, age, education level, illness duration, medical history and family medical history.

### Evaluation of sleep quality

Sleep quality was assessed using the PSQI, which has seven components including subjective sleep quality, sleep latency, sleep duration, habitual sleep efficiency, sleep disturbance, use of sleep medication and daytime dysfunction during the previous month, which are scored on a 4-point rating scale ranging from 0 (none) to 3 (≥ 3 times per week) [27]. In China, a score ≥ 7 has high diagnostic sensitivity and specificity for distinguishing patients with poor sleep from healthy subjects [29]. Total PSQI scores range from 0–21, with a higher score corresponding to poorer sleep quality [27].

### Assessment of depression severity

Depression severity was assessed using the HAMD-17, which comprises 17 items relating to depressed mood, feelings of guilt and suicide, sleep, work and activities [30]. A score < 7 indicates a healthy state, whereas scores of 7–17, 18–24 and > 24 correspond to mild, moderate and severe depression, respectively.

### Cognitive assessment

The MoCA-C is a widely used subjective cognitive screening tool with good reliability and validity for Chinese subjects [31]. It comprises eight dimensions: visual space and executive function, naming, attention, language, abstraction, short-term memory, delayed recall and orientation [32]. The maximum score is 30 points, and a score ≥ 26 indicates normal cognitive function [31].

### Stigma evaluation

The Stigma Scale for Chronic Illness (SSCI) is a 24-item measure of stigma, which evaluates the degree of stigma of chronic neurological diseases and includes 13 internalized (e.g., “I felt embarrassed about my illness”, “Because of my illness, I felt different from others,” etc.) and 11 enacted items (e.g., “Because of my illness, people made fun of me”, “Because of my illness, I was treated unfairly by others”, “I lost friends by telling them that I have this illness”, etc.) [15]. Each item is rated using the following response format: 1 = never, 2 = rarely, 3 = sometimes, 4 = often and 5 = always. The total score ranges from 24 to 120 points and indicates the severity of stigma suffered by the patient. A score < 8 indicates a healthy state, whereas scores > 20 and > 35 correspond to mild and severe stigma, respectively [15]. Internal consistency (Cronbach's alpha value = 0.951), and the Cronbach's alpha of self-stigma and enacted stigma were 0.969 and 0.927.

### Assessment of life quality

The Medical Outcomes Study (MOS) 36-Item Short-Form Health Survey (SF-36) measures health-related quality of life, functioning and well-being and has strong reliability and validity for use in both general and disease-specific populations [33]. All but one of the 36 items are used to score the eight SF-36 scales, which include: Physical Function, Physical Role, Body Pain, General Health, Vitality, Social Function, Emotional Role and Mental Health. It also contains an additional item of Health Transition, which is not part of any dimension and measures the declared evolution of health [34, 35]. Higher scores correspond to better health-related life quality [33]. The Cronbach's alpha cofficients of internal consistency ranged from 0.72 to 0.88, which were satisfactory for group comparison.

### Statistical analysis

SPSS version 20 for windows was used for statistical analyses. Continuous normally distributed data are presented as means ± standard deviations and were evaluated using Student’s *t-*test to compare differences between groups and one-way analysis of variance to determine main effects. The least significant difference test was used for multiple comparisons. Non-normally distributed data are expressed as *P*50 (*P*25 and *P*75), and differences between groups were analysed using the rank-sum test for two independent samples with a completely randomized design (Mann–Whitney *U*). Categorical data were analysed using a chi-squared test. To control for the confounding factors and their influence on the variables, correlations between stigma scores and illness duration (controlling for sex, age and educational level), PSQI score (controlling for sex, age, educational level, illness duration and HAMD-17 score), HAMD-17 score (controlling for sex, age, educational level, illness duration and PSQI score), MoCA-C score (controlling for sex, age, educational level, illness duration, PSQI score and HAMD-17 score) and SF-36 score (controlling for sex, age, educational level, illness duration, PSQI score, HAMD-17 score and MoCA-C score) were assessed using partial correlation analysis. Multiple linear regression was used to explore the correlation between stigma and related influencing factors and identify the contribution of each related influencing factor to changes in stigma. Two-sided *p* values ≤ 0.05 were considered statistically significant.

## Results

### General characteristics of the study subjects

There were no significant differences in sex ratio (C^2^ = 0.8, *p* = 0.4), age (U = 2235.0, *p* = 0.4), or educational level (U = 2840.0, *p* = 0.1) between the two groups (Table 1).

**Table 1:** Demographic characteristics, sleep quality, depression level and cognitive performance of the study subjects

Variable	Chronic insomnia disorder	Healthy controls	Statistic	*P*-value
Number of cases	70	70		
Male/female	21/49	26/44	C^2^ = 0.80	0.4
Age (year)	50.0 (43.0, 55.0)	47.0 (38.5, 54.0)	U = 2235.0	0.4
Education (year)	9.0 (6.0, 12.0)	9.0 (8.8, 15.0)	U = 2840.0	0.1
Illness duration (year)	6.0 (3.0, 13.5)			
PSQI score	16.0 (14.0,17.0)	4.0 (3.0, 5.0)	U = 2485.0	< 0.001
HAMD-17 score	11.0 (7.8, 13.0)	2.0 (0.0, 3.0)	U = 69.5	< 0.001
MoCA-C score	23.0 (20.0, 26.0)	26.0 (26.0, 28.0)	U = 3997.5	< 0.001

Normally distributed variables are shown as means ± standard deviations; non-normally distributed variables are shown as P50 (P25, P75)

*Abbreviations*: *HAMD-17* 17-item Hamilton Depression Rating Scale, *MoCA-C* Chinese-Beijing Version of Montreal Cognitive Assessment, *PSQI* Pittsburgh Sleep Quality Index

### Sleep quality and depression severity

PSQI scores differed significantly between groups (U = 2485.0, *p* < 0.001), and the CID group had significantly higher scores than the control group. HAMD-17 scores differed significantly between groups (U = 69.5, *p* < 0.001); and the score of the CID group was almost six times higher than that of the health controls (Table 1).

### Cognitive function

There were significant differences in MoCA-C scores between the two groups (U = 3997.5, *p* < 0.001). Patients with CID had significantly lower total MoCA-C scores (23.0 [20.0, 26.0]) than controls (26.0 [26.0, 28.0]; Table 1).

### Stigma scores

The ratio of stigma was significantly different between the two groups (C^2^ = 35.6, *p* < 0.001). For performance in stigma, significantly higher scores were observed in patients than controls for the items of total (U = 662.0, *p* < 0.001), internalized (U = 593.0, *p* < 0.001) and enacted (U = 1568.0, *p* < 0.001) stigma scores (Table 2).

**Table 2:** Stigma and life quality of the study subjects

Terms		Chronic insomnia disorder	Healthy controls	Statistic	*P*-value
Stigma (n, %)		57 (81.4)	22 (31.4)	C^2^ = 35.6	< 0.001
SSCI score	Total	34.5 (26.0, 42.3)	24.0 (24.0, 24.0)	U = 662.0	< 0.001
	Internalized	21.0 (14.0, 29.0)	13.0 (13.0, 13.0)	U = 593.0	< 0.001
	Enacted	12.0 (11.0, 13.0)	11.0 (11.0, 12.0)	U = 1568.0	< 0.001
SF-36 score	Physical Function	90.0 (85.0, 100.0)	95.0 (85.0, 100.0)	U = 2164.0	0.2
	Physical Role	50.0 (25.0, 100.0)	100.0 (50.0, 100.0)	U = 1560.5	< 0.001
	Body Pain	74.0 (62.0, 84.0)	84.0 (74.0, 100.0)	U = 1633.5	< 0.001
	General Health	52.1 ± 22.5	72.9 ± 18.8	U = 1194.0	< 0.001
	Vitality	55.0 (40.0, 75.0)	80.0 (70.0, 85.0)	U = 1169.5	< 0.001
	Social Function	77.8 (55.6, 100.0)	88.9 (77.8, 100.0)	U = 1703.0	0.001
	Emotional Role	66.7 (0.0, 66.7)	100.0 (66.7, 100.0)	U = 1451.5	< 0.001
	Mental Health	48.0 (36.0, 72.0)	76.0 (67.0, 84.0)	U = 1147.0	< 0.001
	Health Transition	25.0 (25.0, 50.0)	50.0 (50.0, 50.0)	U = 1341.0	< 0.001

Normally distributed variables are shown as means ± standard deviations; non-normally distributed variables are shown as P50 (P25, P75)

*Abbreviations*: *SF-36* Medical Outcome Study 36-Item Short-Form Health Survey, *SSCI* Stigma Scale for Chronic Illness

### Life quality levels

There were significant intergroup differences for the SF-36 items of Physical Role (U = 1560.5, *p* < 0.001), Body Pain (U = 1633.5, *p* < 0.001), General Health (U = 1194.0, *p* < 0.001), vitality (U = 1169.5, *p* < 0.001), Social Function (U = 1703.0, *p* = 0.001), Emotional Role (U = 1451.5, *p* < 0.001), Mental Health (U = 1147.0, *p* < 0.001) and Health Transition (U = 1341.0, *p* < 0.001; Table 2).

### Correlations between stigma scores and illness duration and possible factors

In the CID group, partial correlation analysis showed that illness duration was positively correlated with total (*r* = 0.62, *p* < 0.001), internalized (*r* = 0.56, *p* < 0.001) and enacted stigma scores (*r* = 0.51, *p* < 0.001). For the correlations between stigma score and PSQI score (controlling for sex, age, educational level, illness duration and HAMD-17 score) and HAMD-17 (controlling for sex, age, educational level, illness duration and PSQI score), results showed that total and internalized stigma scores were positively correlated with PSQI (*r* = 0.28, *p* = 0.02; *r* = 0.32, *p* = 0.01) and HAMD-17 (*r* = 0.50, *p* < 0.001; *r* = 0.53, *p* < 0.001) scores, respectively. Additionally, total stigma scores were negatively correlated with the SF-36 items of Vitality (*r* =  − 0.27, *p* = 0.03) and Mental Health (*r* =  − 0.41, *p* = 0.001), after controlling for sex, age, education and MoCA-C scores (Table 3). Significant negative correlations were found between internalized stigma and the SF-36 items of Physical Role (*r* =  − 0.27, *p* = 0.03), Vitality (*r* =  − 0.33, *p* = 0.001) and Mental Health (*r* =  − 0.45, *p* < 0.001).

**Table 3:** Partial correlations among stigma scores and illness duration and scores of PSQI, HAMD-17, MoCA-C and SF-36 in patients with CID

Variable		Stigma		
		Total	Internalized	Enacted
Illness duration^a^ (year)		0.62^#^	0.56^#^	0.51^**^
PSQI^b^ score		0.30^*^	0.34^#^	0.10
HAMD-17^c^ score		0.45^#^	0.48^#^	0.10
MoCA-C^d^ score		− 0.06	− 0.08	0.004
SF-36^e^ score	Physical Function	− 0.09	− 0.10	− 0.02
	Physical Role	− 0.22	− 0.27^*^	− 0.02
	Body Pain	− 0.05	− 0.17	0.18
	General Health	− 0.08	− 0.14	0.03
	Vitality	− 0.27^*^	− 0.33^#^	− 0.04
	Social Function	− 0.14	− 0.15	− 0.06
	Emotional Role	− 0.06	− 0.09	0.01
	Mental Health	− 0.41^#^	− 0.45^#^	− 0.19
	Health Transition	0.12	0.13	0.14

*Notes*: ^a^controlling for sex, age, and educational level; ^b^controlling for sex, age, educational level, illness duration and HAMD-17; ^c^controlling for sex, age, educational level, illness duration and PSQI; ^d^controlling for sex, age, educational level, illness duration, PSQI and HAMD-17; ^e^controlling for sex, age, educational level, illness duration, PSQI, HAMD-17 and MoCA-C. *p < 0.05. ^#^*p* < 0.01

*Abbreviations*: *HAMD-17* 17-item Hamilton Depression Rating Scale, *PSQI* Pittsburgh Sleep Quality Index, *SF-36* Medical Outcome Study 36-Item Short-Form Health Survey, *CID* chronic insomnia disorder

### Multiple linear regression between stigma scores and related factors

For the regression analysis in the CID group, scores for the different items of stigma were defined as the dependent variable and those for all related factors were defined as independent variables based on the partial correlation analysis. Results revealed a significant linear regression, in which illness duration was independently positively correlated with total (β = 0.433, *p* < 0.001), internalized (β = 0.326, *p* < 0.001) and enacted (β = 0.441, *p* < 0.001) stigma scores. The SF-36 item of Mental Health was independently negatively correlated with total (β =  − 0.346, *p* = 0.007) and internalized (β =  − 0.377, *p* = 0.004) stigma scores (Table 4).

**Table 4:** Multiple linear regression between stigma and related influencing factors in patients with CID

Variable		Total Stigma				Internalized Stigma				Enacted Stigma			
		Standardized *β*	*t*	*P*	B (95% CI)	Standardized *β*	*t*	*P*	B (95% CI)	Standardized *β*	*t*	*P*	B (95% CI)
Illness duration (year)		0.433	5.113	< 0.001	0.357–0.816	0.326	3.760	< 0.001	0.161–0.527	0.441	3.841	0.000	0.104–0.329
PSQI score		0.062	0.692	0.492	− 0.457–0.942	0.086	0.935	0.354	− 0.297–0.818	0.035	0.285	0.777	− 0.295–0.392
HAMD-17 score		0.127	1.325	0.190	− 0.192–0.946	0.146	1.492	0.141	− 0.115–0.792	− 0.048	− 0.366	0.716	− 0.330–0.228
SF-36 score	Physical Role	− 0.128	− 1.484	0.143	− 0.079–0.012	− 0.149	− 1.690	0.096	− 0.067– 0.006	− 0.050	− 0.429	0.669	− 0.027–0.018
	Vitality	0.002	0.013	0.989	− 0.120–0.122	− 0.015	− 0.122	0.903	− 0.102–0.090	0.086	0.522	0.604	− 0.044–0.075
	Mental Health	− 0.346	− 2.775	0.007	− 0.296–0.048	− 0.377	− 2.952	0.004	− 0.244–0.047	− 0.278	− 1. 645	0.105	− 0.111–0.011

*Abbreviations*: *HAMD-17* 17-item Hamilton Depression Rating Scale, *PSQI* Pittsburgh Sleep Quality Index, *SF-36* Medical Outcome Study 36-Item Short-Form Health Survey, *CID* chronic insomnia disorder, *CI* confidence interval

## Discussion

Patients with chronic insomnia are associated with greater depressive symptoms and cognitive impairment [36, 37]. Different levels of depression and cognitive impairment will further exacerbate insomnia [38, 39]. Stigma as a particularly burdensome personal and social challenge is closely related to psychiatry disorders, such as depression [40, 41]. Additionally, several severe physical disorders are also accompanied by stigmatization [42–46]. Although 50–80% of individuals with a chronic disease experience stigma at varying levels [47, 48], little is known about the extent to which stigma impacts chronic insomnia.

### CID patients have an increased risk of stigma

The existence of stigma could reduce patients’self-esteem and treatment compliance, resulting in social avoidance behavior and maladaptation, which manifests as poor sleep quality indirectly [45, 49–51]. In the present study, we found that compared with healthy subjects, CID patients had higher stigma ratios and scores. This indicated that CID patients have some degree of stigma, and the level of stigma is significantly higher than that of controls. Given the correlation of chronic insomnia with greater depressive symptoms and stigma with depressive symptoms has been consistently confirmed in previous research [52], we proposed that such interaction—among stigma, psychiatry disorders, and chronic insomnia—leads to the existence of stigma in patients with chronic insomnia disorder. Additionally, researches show that stigma could also exert a negative influence on sleep due to increased negative self-thought and activation of the ruminative thought process [53, 54]. Indeed, individuals with severe sleep problems are more likely to have depression, which may further exacerbate the severity of stigma in CID patients [55]. Considering the relationship between chronic insomnia and stigma is still at the preliminary exploration stage and we are the first relatively comprehensive research about stigma in patients with chronic insomnia disorder, further studies are needed to provide theoretical approval to reach a definitive conclusion between them.

### Relationship between stigma scores and related influencing factors

The occurrence of stigma is related to several influencing factors, such as illness duration, life quality, mental health, etc. [52]. Additionally, with a prolonged disease course, patients under chronic disease conditions exhibit different levels of stigma [56–60]. In our study, both the partial correlation and multiple linear regression analyses showed that the longer the illness duration, the higher the stigma score, which is in line with previous studies [52]. To gain a comprehensive understanding of stigma changes, it would be meaningful to further observe these populations in future studies. Given that stigma is influenced not only by illness duration [61, 62], and identifying other influencing factors is necessary.

Life quality is a critical factor that promotes the progression of stigma [63]. As common comorbidity disease of chronic insomnia, individuals with serious mental illness die prematurely by decades, which is not driven by increased suicides or injuries but by poor physical health, which is influenced by stigma [64]. Our results showed that stigma scores were negatively correlated with most of the subitems of the SF-36, which suggested that stigma in CID patients is related to health quality. Interestingly, the multiple linear regression analysis showed that only the Mental Health item of the SF-36 was an independent risk factor of stigma, which further demonstrates the influence of Mental Health on stigma [16, 17]. In other words, mental illness severity should be considered a predictor of stigma among people with psychiatry disorders [65, 66]. This may be because psychiatric disorders, such as depression, affect the perception of the circumstances of daily life [67].

In individuals with psychiatric disorders, stigma may represent a potent stressor that disrupts sleep and impairs health and quality of life [68]. Furthermore, via the content and process of stigma, discrimination has a significant indirect effect on sleep disturbance in these individuals [53]. In the present study, the partial correlation analysis in the CID patients revealed that poor sleep quality and severe depression were positively correlated with different aspects of stigma, such as total and internalized stigma. However, these correlations were not observed in the subsequent multiple linear regression analysis. These results suggest that in CID patients, the effect of stigma on sleep quality and depression severity is mainly dependent on illness duration and life quality, especially mental health.

### Lower life quality in CID patients

Life quality is a major outcome variable in the evaluation of alternative treatments for sleep disorders [69]. Poor-quality sleep can negatively affect an individual’s subjective well-being and quality of life [70]. Particularly under pathological conditions, high quality of life positively affects sleep [71]. Consistent with previous research, we also found that CID patients had significantly lower scores than controls in most domains of the SF-36, which included Physical Role, Body Pain, General Health, Vitality, Social Function, Emotional Role, Mental Health and Health Transition, which suggests that CID negatively impacts quality of life [70, 71]. However, we did not find a significant difference in the item of Physical Function between the two groups. Even in the partial correlation analysis, the item of Physical Function did not correlate with any of the domains of stigma. Such a discrepancy may be attributed to several factors, such as ethnic origin, sex, comorbidities and differences in the severity of insomnia [72–74]. Further studies are required to reach a definitive conclusion.

## Limitations

The current study had several limitations. First, the findings that patients with chronic insomnia had poorer sleep quality, greater depressive symptoms and poorer quality of life have been consistently found in previous literature. Previous research has also found the finding on the correlation of stigma with depressive symptoms and illness duration. The study will be more interesting if it examines prospective predictors of the stigma or prospective outcomes associated with insomnia. Second, the measure used to measure stigma was a measure designed for neurological disease/non-specific chronic illnesses, not specific to the experience of stigma related to insomnia. Given that the relationship between stigma and insomnia is still at the preliminary exploration stage, a specific evaluation measure to the experience of stigma related to insomnia is urgently needed if it could examine more insomnia-specific stigma. Third, the recruited insomnia subjects were all come from the hospital, which may lead to the potential bias of their stigma scores. Additionally, the level of stigma is affected by numerous other variables rather than the aspects that we listed only. Therefore, other influence factors ought to be considered in future research.

## Conclusion

Patients with CID had an increased risk of stigma, suggesting that different levels of stigma underlie CID. Additionally, illness duration and Mental Health (an item in SF-36) were the main factors related to stigma. CID patients who have long illness duration or Mental Health problems ought to seek professional help timely. Caregivers should prioritize monitoring CID patients living with high levels of stigma and developing targeted interventions to eliminate the stigmatization of this group. In future, further studies are needed to validate whether successful treatment of insomnia can relieve stigma.

## Supplementary Information

**Additional file 1.**

## Data Availability

The datasets are presented in the additional supporting files and are available from the corresponding author (doctorcgh@163.com) or the editor at any request.

## Abbreviations

BMI: Body mass index
CID: Chronic insomnia disorder
CON: Healthy controls
HAMD-17: 17-Item Hamilton Depression Rating Scale
HIV: Human immunodeficiency virus
ICSD-3: International Classification of Sleep Disorders, Third Edition
MoCA: Montreal Cognitive Assessment
MoCA-C: Chinese-Beijing Version of Montreal Cognitive Assessment
MOS: Medical outcomes study
MS: Multiple sclerosis
PSQI: Pittsburgh Sleep Quality Index
SF-36: Mos 36-Item Short-Form Health Survey
SSCI: Stigma Scale for Chronic Illness
